# Supplementary material for: The genome sequence of the Timothy Tortrix, Aphelia paleana (Hubner, 1793) (Lepidoptera: Tortricidae)
Source: Wellcome Open Res. 2026 Sep 24;11:733. [Version 1] doi: 10.12688/wellcomeopenres.27914.1 (PMC13684024; doi:10.12688/wellcomeopenres.27914.1)
Supplement: Peer review history

# Reviewer report: Reviewer response for version 1

**DOI:** 10.21956/wellcomeopenres.30714.r172120

**Published:** 2026-10-20

**Authors:** Tracy Liesenfelt

**Affiliations:** 1 University of Florida, Gainesville, Florida, USA

The authors report a genome note for the assembly of *Aphelia paleana*, the Timothy Tortrix, as part of the larger efforts of the Darwin Tree of Life Project. A brief background of the species description and habitat is provided alongside a robust and detailed methodology of the sequencing and assembly process. This genome assembly reports 30 chromosomes including the Z chromosome with high assembly quality metrics including a k-mer completeness score of 99.31% for the combined haplotypes and a BUSCO score of 98.5% of the expected gene set. Both the data and protocols used are well documented and available.

 I do have a few minor comments and suggestions.

 First, a small suggestion for the introduction, since this species is described as a micro-moth, I think it could be beneficial for the reader to include an average size for adults of this species.

 Next, a minor comment in part of the Methods section: “Sample acquisition and DNA barcoding." In this paragraph, it states “A small sample was dissected from the specimen and stored in ethanol," The language here is a little vague, and I think further description on which body parts are being stored in ethanol could be beneficial. Based on the protocol cited here, I assume this is referring to the legs taken for DNA barcoding, but further clarity in this genome note would be beneficial to replication of these methods.

 Another note is there are some minor inconsistencies within the formatting of the Methods. Within the body text, there is variation between sections on the level of detail being included. For example, in the “Nucleic acid extraction section” when referring to the usage of a Qubit for quality metrics, it is referred to as “a Qubit Fluorometer using the Qubit dsDNA High Sensitivity Assay kit.” However in the following section “PacBio HiFi library preparation and sequencing” when Qubit is mentioned again, it is listed as “a Qubit Fluorometer v4.0 (Thermo Fisher Scientific) and the Qubit 1X dsDNA HS assay kit” now providing a version number and company when mentioned. I think this formatting should either be provided at first mention of a technology or used throughout.

 Along with this, throughout the text some assembly tools are accompanied by their version numbers while others aren't, but when they are mentioned, the formatting varies in either stating "version #, v# or v.#." I do however acknowledge if not provided in text, the authors provide a more expansive list of all of the software versions for each tool in Table 5.

Are the datasets clearly presented in a usable and accessible format, and the assembly and annotation available in an appropriate subject-specific repository?

Yes

Are sufficient details of the sequencing and extraction, software used, and materials provided to allow replication by others?

Yes

Are the rationale for sequencing the genome and the species significance clearly described?

Yes

Are the protocols appropriate and is the work technically sound?

Yes

Reviewer Expertise:

insects: Hemiptera, genomics, population genetics, genome assembly

I confirm that I have read this submission and believe that I have an appropriate level of expertise to confirm that it is of an acceptable scientific standard.

# Reviewer report: Reviewer response for version 1

**Published:** 2026-09-24

**Authors:** Automated Benchmarking

**Affiliations:** 1 Wellcome Sanger Institute, Hinxton, UK

As this article is a Genome Note, Automated Benchmarking has been used to determine whether the reported assembly passes four defined checks established by the Earth BioGenome Project: the raw and assembled data are publicly available, the Earth BioGenome Project three-part metric is met, the k-mer completeness exceeds 90%, and the BUSCO score passes the stated threshold. For Aphelia paleana (ilAphPale1), all four checks passed. Data available: pass. ENA study PRJEB78812, raw-read accessions ERR13485752 and ERR13494009, and assembly accessions GCA_964237545.1 and GCA_964237565.1 are public. Earth BioGenome Project three-part metric: pass. Haplotype 1 is reported as 6.C.Q66, meeting the 6.C.Q40 reference standard. k-mer completeness: pass. The combined haplotype assemblies have 99.31% k-mer completeness, exceeding the Earth BioGenome Project target of 90%. BUSCO: pass. Haplotype 1, which is proposed as the reference assembly for this species, has 98.5% complete BUSCOs (98.1% single-copy and 0.5% duplicated). BUSCO software version: 5.5.0; lineage dataset: lepidoptera_odb10. It meets the target of greater than 90% complete and single-copy BUSCOs. This decision uses values reported in the Genome Note and public accession records.

Reviewer Expertise:

NA

I confirm that I have read this submission and believe that I have an appropriate level of expertise to confirm that it is of an acceptable scientific standard.
